# Early Pregnancy Nutritional Adequacy and Subsequent Gestational Diabetes Risk by Body Mass Index: A Prospective Cohort Study of 2227 Korean Women

**DOI:** 10.3390/nu17223569

**Published:** 2025-11-14

**Authors:** Hye-Ji Han, Hyun Jung Lee, Jin Woo Kim, Su Ji Yang, Ju Yeon Kim, Yong Jun Choi, Seoyeon Kim, Nari Kim, Young Ran Kim, Sang Hee Jung, Ji Hyon Jang, Youjeong Hwang, Min Hyoung Kim, Moon Young Kim, Ji Hyae Lim, Hyun Mee Ryu

**Affiliations:** 1 Department of Obstetrics and Gynecology, CHA University Bundang Medical Center, Seongnam 13496, Republic of Korea; 2 Smart MEC Healthcare R&D Center, CHA Bundang Medical Center, Seongnam 13520, Republic of Korea; 3 Inseong Medical Foundation Hallym Hospital, Incheon 21079, Republic of Korea; 4 Department of Obstetrics and Gynecology, MizMedi Hospital, Seoul 07639, Republic of Korea; 5 Department of Obstetrics and Gynecology, CHA Gangnam Medical Center, Seoul 06135, Republic of Korea

**Keywords:** diabetes, gestational, nutritional status, pregnancy, nutritional requirements, risk factors

## Abstract

**Background/Objectives:** This study evaluated the association between nutrient intake adequacy during early pregnancy and gestational diabetes mellitus (GDM) risk through a prospective cohort study of Korean pregnant women. **Methods:** A total of 2227 singleton pregnant women were included in this study. Dietary assessment was conducted once during early pregnancy enrollment using the 24 h dietary recall method. The collected dietary data were analyzed using the CAN-Pro Korean food composition database to calculate nutrient-specific intake levels. Nutrient Adequacy Ratio (NAR) for each nutrient and Mean Adequacy Ratio (MAR), which integrates individual NARs, were calculated based on intake levels. GDM diagnosis was determined using oral glucose tolerance test (OGTT) results conducted between 24 and 28 weeks of gestation. Relative risk (RR) for each individual nutrient was calculated, and logistic regression analysis was performed to estimate odds ratios (OR) for GDM risk according to MAR quartiles. **Results:** Among 2227 participants, 157 women developed GDM. Compared to the highest MAR quartile, women in the lowest quartile showed significantly increased GDM risk (OR = 1.82, 95% CI: 1.10–2.99), with the second lowest quartile demonstrating similarly elevated risk (OR = 1.75, 95% CI: 1.06–2.88). Among individual nutrients, inadequate vitamin D intake was associated with the highest GDM risk (RR = 3.84), followed by insufficient intakes of vitamin K (RR = 1.89), vitamin B_6_ (RR = 1.62), niacin (RR = 1.54), and calcium (RR = 1.39). Body mass index-stratified analysis revealed that the association between low nutritional adequacy and GDM risk was particularly pronounced in women with BMI ≥ 25.0 kg/m^2^, showing up to a four-fold increased risk in the lowest adequacy groups. **Conclusions**: These findings suggest that low overall dietary adequacy and nutritional imbalance during early pregnancy are associated with increased GDM risk. The results underscore the importance of ensuring adequate and balanced nutrition during early pregnancy for GDM prevention.

## 1. Introduction

Pregnancy represents a unique physiological state characterized by substantially increased nutritional requirements, making balanced nutrient intake essential for both fetal growth and development as well as maternal health maintenance [1,2]. Adequate nutritional provision during pregnancy reduces the risk of neural tube defects, low birth weight, and preterm birth, while also playing a crucial role in preventing maternal pregnancy complications [3]. Gestational diabetes mellitus (GDM) is defined as glucose intolerance of any degree first recognized during pregnancy, regardless of the severity of hyperglycemia [4]. Due to the special circumstances of pregnancy, pharmacological treatment options are limited, making dietary intervention and lifestyle modification the primary and safest approaches for both treatment and prevention [3]. Consequently, dietary factors play a pivotal role in the development and progression of GDM.

The global prevalence of GDM continues to rise, driven by increasing maternal age and body mass index (BMI). According to the International Diabetes Federation, approximately 14% of pregnancies worldwide are affected by GDM [5]. In Korea, data from the Health Insurance Review and Assessment Service reveal that GDM prevalence increased 4.8-fold from 4.6% to 22.3% between 2010 and 2023, demonstrating a persistent and significant upward trend [6]. Recent studies have reported that early GDM, diagnosed before 20 weeks of gestation, is associated with more severe maternal and fetal complications compared to GDM diagnosed at the conventional 24–28 week timepoint [7,8]. These findings underscore the growing importance of adequate nutritional management and glycemic control from early pregnancy.

Although consensus on effective dietary approaches for GDM prevention and management remains elusive, recent studies have proposed several strategies. The Mediterranean diet has demonstrated approximately a 36% reduction in GDM risk [9], while low glycemic index/glycemic load approaches have shown effectiveness in glycemic control and reducing macrosomia risk [10]. However, these dietary pattern-focused studies have shown inconsistent results across different populations [11,12,13,14]. Emerging evidence suggests that overall nutritional adequacy, rather than specific dietary patterns, may be critical for pregnancy outcomes. Higher diet quality during early pregnancy was associated with reduced risk of fetal growth restriction in Spanish women [15] and decreased risk of small-for-gestational-age infants in Korean women [16]. However, research specifically examining the relationship between nutritional adequacy and GDM remains limited, particularly in non-Western populations. Existing dietary quality indices, such as the Healthy Eating Index (HEI) [17] and the Dietary Approaches to Stop Hypertension (DASH) score [18], are predominantly food-based assessment tools that evaluate diet quality based on specific food groups and their consumption frequencies. This food-centric approach may not adequately capture nutritional adequacy in populations with diverse dietary compositions, suggesting that nutrient-based evaluation methods should serve as a foundational framework for assessing dietary quality across different populations [19].

The Nutrient Adequacy Ratio (NAR) and Mean Adequacy Ratio (MAR) offer a nutrient-based approach for standardized, quantitative assessment of dietary adequacy across diverse populations. These methods allow individualized evaluation based on person-specific requirements that vary by age, sex, and physiological status, such as pregnancy [20]. Therefore, this study has three objectives. First, we evaluate the extent to which Korean pregnant women adhere to recommended nutrient intakes. Second, we quantify nutrient intake relative to recommendations using NAR and MAR and examine associations between nutritional adequacy ratios and GDM incidence. Third, we provide foundational evidence for personalized nutrition approaches to GDM prevention through BMI-stratified analysis.

## 2. Materials and Methods

### 2.1. Study Design and Population

**Figure 1:**
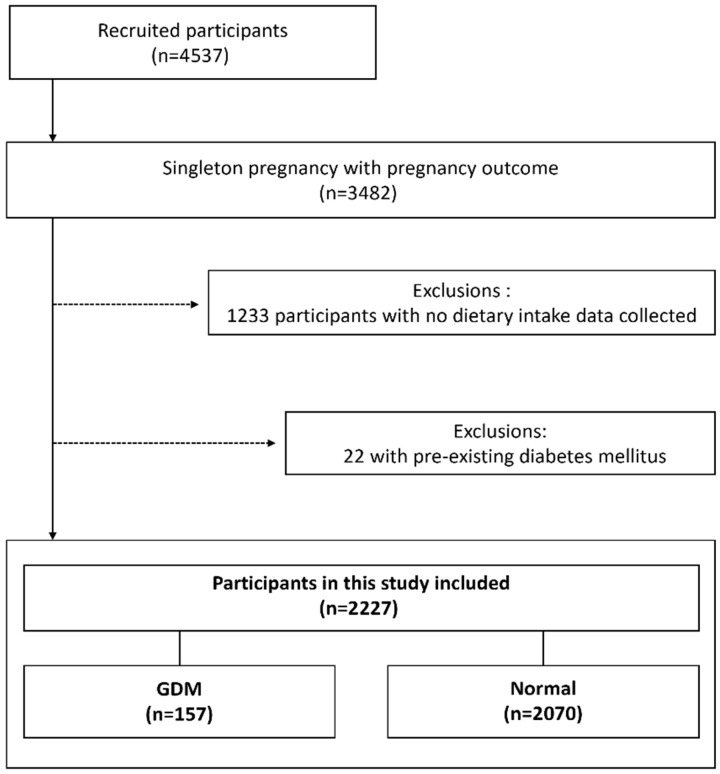
Study Population Flow Chart. GDM, gestational diabetes mellitus.

Data for this study were obtained from the Korean Pregnancy Outcome Study (KPOS) [21], a longitudinal prospective cohort study designed to investigate adverse pregnancy outcomes, which was conducted at two secondary hospitals in Seoul, Korea, between March 2013 and January 2017. Of the 4537 women enrolled in the cohort, 2227 singleton pregnancies were included in the present analysis, after excluding women with incomplete dietary assessment data (*n* = 1233) and pre-existing diabetes mellitus (*n* = 22). Study participants visited the participating hospitals during the first trimester of pregnancy, at which time baseline demographic and clinical characteristics were collected along with dietary assessment data. GDM was diagnosed using a two-step screening approach. Routine screening with a 50 g glucose challenge test (GCT) was conducted between 24 and 28 gestational weeks. Participants with GCT results ≥140 mg/dL underwent a confirmatory oral glucose tolerance test (OGTT) for definitive GDM diagnosis. The detailed diagnostic criteria and protocol for GDM in the KPOS are described in a previous study [21]. Based on the diagnostic results, participants were categorized into two groups: Normal (*n* = 2070) and GDM (*n* = 157) (Figure 1). This study was conducted in accordance with the Declaration of Helsinki and approved by the Institutional Review Board of the CHA Bundang Medical Center (CHAMC 2021-04-052).

### 2.2. Dietary Assessment

Dietary intake assessment was performed during the first trimester of pregnancy (8–12 weeks of gestation) by trained registered dietitians using standardized protocols. A single 24 h dietary recall method was employed during one-on-one interviews with participants. This method has been validated for use in Korean populations and provides reliable estimates of usual dietary intake when conducted by trained professionals [22]. During the interview, participants were asked to recall all foods and beverages consumed during the previous 24 h period, including detailed information about food preparation methods, portion sizes, and timing of consumption. Visual aids, including food models, measuring cups, and portion size guides, were used to enhance accuracy of portion size estimation. The collected dietary data were analyzed using the CAN-Pro (Computer Aided Nutritional Analysis Program) version 4.0 [23], which contains comprehensive nutrient composition data for Korean foods based on the Korean Food Composition Database. This program calculated individual nutrient intakes for each participant. To ensure data quality and minimize the influence of implausible values, extreme outliers in nutrient intake data were addressed using winsorization at the 5th and 95th percentiles.

### 2.3. Nutritional Adequacy Assessment

Nutritional adequacy was evaluated using the NAR and MAR methods, which are established tools for assessing overall dietary quality [24]. We calculated the NAR as a percentage for 13 nutrients with established Recommended Dietary Intake (RDI), including carbohydrates, protein, vitamin A, vitamin C, niacin, vitamin B6, folate, vitamin B12, calcium, magnesium, iron, zinc, and iodine. NAR was calculated using the following formula: NAR = (Individual daily intake/Recommended daily intake). The NAR for a specific nutrient represents the ratio of a participant’s intake to the RDI for that nutrient, with a maximum value of 1.0. An NAR below 1.0 indicates that nutrient intake is below the RDI, while an NAR equal to 1.0 indicates that nutrient intake meets or exceeds the RDI [24]. To evaluate overall dietary adequacy, the MAR was calculated as the average of the 13 NARs using the following formula: MAR = (Sum of truncated NARs for all nutrients)/(Total number of nutrients). This study used the 2020 Korean Dietary Reference Intakes [25]. The definitions and abbreviations of nutritional assessment terms used in this study are presented in Table 1.

### 2.4. Covariate Assessment

Comprehensive information on potential confounding variables was collected through standardized questionnaires and medical record reviews. Maternal characteristics included age, educational level (high school, college, graduate), household income (<4 million Korean won per month, ≥4 million Korean won per month), and employment status. Lifestyle factors assessed included physical activity levels using the International Physical Activity Questionnaire (IPAQ) [26], alcohol consumption, smoking status (never, pre-pregnancy only, continued during pregnancy), and dietary supplement use. Physical activity was categorized as moderate-to-vigorous physical activity (MVPA) ≥150 min per week versus <150 min per week. Pre-pregnancy weight and height were self-reported, and body mass index (BMI) was calculated and categorized according to Asian-specific cutoffs: underweight (<18.5 kg/m^2^), normal (18.5–22.9 kg/m^2^), overweight (23.0–24.9 kg/m^2^), and obese (≥25.0 kg/m^2^) [27]. Medical historical variables included parity (nulliparous vs. parous), history of diabetes mellitus, hypertension, and severity of nausea and vomiting during pregnancy (none, mild, or severe). These factors were included as potential confounders based on previous literature documenting their associations with both nutritional status and GDM risk.

### 2.5. Statistical Analysis

Statistical analyses were performed using SPSS version 28.0 (IBM Corp., Armonk, NY, USA). Descriptive statistics were calculated for all variables, with continuous variables presented as mean ± standard deviation and categorical variables as frequencies and percentages. Extreme outliers in nutrient intake data were addressed using historization at the 5th and 95th percentiles to minimize the influence of implausible values. Participants were divided into quartiles based on their MAR values to examine dose–response relationships. Baseline characteristics across MAR quartiles were compared using one-way analysis of variance (ANOVA) for continuous variables and chi-square tests for categorical variables.

The association between MAR quartiles and GDM risk was examined using logistic regression analysis. Covariates for adjustment were selected based on variables showing significant differences between the GDM and normal groups in bivariate analyses (Appendix A Table A1) and established risk factors from previous literature. Model 1 adjusted for established GDM risk factors, including maternal age, pre-pregnancy BMI, employment status, parity, and hypertension. Model 2 included additional adjustments for macronutrient composition (carbohydrate, protein, and saturated fat as percentages of total energy intake) and dietary fiber intake to account for overall dietary patterns. Trend tests were performed by including the median MAR value of each quartile as a continuous variable in the logistic regression models.

BMI-stratified analyses were performed after confirming significant interaction between MAR and pre-pregnancy BMI (*p* for interaction < 0.05). Associations between MAR quartiles and GDM risk were examined separately within the BMI < 23.0 kg/m^2^, BMI 23.0–24.9 kg/m^2^, and BMI ≥ 25.0 kg/m^2^ categories using the same multivariable adjustment strategy. For individual nutrients, relative risks and 95% confidence intervals were calculated comparing women with inadequate intake (below EAR or AI) versus those with adequate intake. Chi-square tests were used to compare the prevalence of inadequate intake between women who developed GDM and those who did not. All tests were two-tailed, and statistical significance was set at *p* < 0.05.

### 2.6. Use of Artificial Intelligence

During the preparation of this manuscript, the authors used Claude 4.5 (Anthropic) for English language editing and grammar checking. The authors reviewed and edited the output and take full responsibility for the content of this publication

## 3. Results

### 3.1. Study Population Characteristics

Among 2227 pregnant women, 157 (7.0%) developed GDM (Figure 1). Baseline characteristics showed significant differences across MAR quartiles. Higher nutritional adequacy was associated with substantially higher energy intake, ranging from approximately 1328 kcal/day in the lowest quartile to 2104 kcal/day in the highest quartile. Women with higher MAR also had higher educational attainment and greater pre-pregnancy physical activity. GDM prevalence showed a decreasing trend from 8.8% in the lowest to 5.2% in the highest quartile (Table 2). Compared to women without GDM, those who developed GDM were older (mean age 35.0 vs. 33.3 years), more frequently obese (31.2% vs. 9.7%), more often multiparous (51.0% vs. 40.3%), and had lower rates of balanced dietary intake (41.4% vs. 52.8%) (Appendix A Table A1).

### 3.2. Association Between Overall Nutritional Adequacy and GDM Risk

Table 3 presents the associations between MAR quartiles and GDM incidence. In the fully adjusted model, women in the lowest MAR quartile had 1.82 times higher odds of developing GDM compared to the highest quartile (OR = 1.82, 95% CI: 1.10–2.99, *p* for trend = 0.012). The second quartile also showed elevated risk (OR = 1.75, 95% CI: 1.06–2.88), while the third quartile demonstrated a non-significant trend. These associations remained consistent after adjusting for age, pre-pregnancy BMI, employment status, parity, hypertension, and macronutrient composition.

### 3.3. Individual Nutrient Inadequacies and GDM Risk

Table 4 present individual nutrient intakes and their associations with GDM risk. Women who developed GDM had significantly lower mean intakes of several key nutrients, including vitamin B_6_, vitamin C, magnesium, and potassium. The prevalence of inadequate intake was notably high, with over 90% having insufficient vitamin D (98.1% in GDM vs. 92.7% in normal group) and nearly all participants showing inadequate magnesium intake. Other nutrients with high inadequacy rates included iron (approximately 86%), dietary fiber (approximately 77%), and potassium (76–82%). Several nutrient inadequacies showed significant associations with increased GDM risk. Vitamin D inadequacy demonstrated the strongest association (RR = 3.84, 95% CI: 1.24–11.90), followed by vitamin K (RR = 1.89), vitamin B_6_ (RR = 1.62), niacin (RR = 1.54), and calcium (RR = 1.39, all *p* < 0.05). Folate and vitamin C showed borderline significance.

### 3.4. BMI-Stratified Analysis

Table 5 presents the associations between MAR and GDM risk stratified by pre-pregnancy BMI categories. The relationship between nutritional adequacy and GDM risk varied significantly across BMI groups. Among women with BMI < 23.0 kg/m^2^, those in the lowest MAR quartile had nearly twice the odds of developing GDM compared to the highest quartile (OR = 1.96, 95% CI: 1.01–3.79, *p* for trend = 0.031). In women with BMI 23.0–24.9 kg/m^2^, no significant association was observed. However, women with BMI ≥ 25.0 kg/m^2^ demonstrated the strongest associations, with the second lowest MAR quartile showing the highest risk (OR = 4.20, 95% CI: 1.30–13.50), followed by the lowest quartile (OR = 3.17) and third quartile (OR = 2.64), with a significant trend (*p* = 0.037).

**Figure 2:**
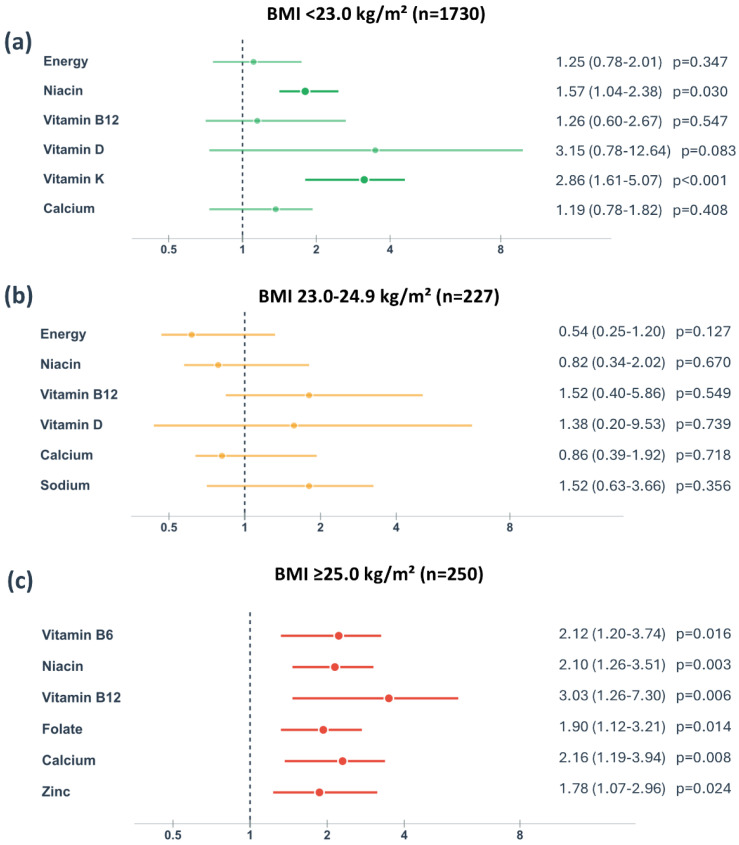
Relative risk of gestational diabetes mellitus associated with inadequate nutrient intake stratified by pre-pregnancy body mass index categories. BMI, body mass index. Data are presented as relative risk with 95% confidence intervals for nutrient intake below the Estimated Average Requirement (EAR) or Adequate Intake (AI) when EAR is not established. The vertical dashed line represents relative risk = 1.0 (no association). Statistical significance was determined using chi-square tests (*p* < 0.05).

To further examine whether these associations varied by BMI status, we conducted a stratified analysis of individual nutrient inadequacies. The forest plot (Figure 2) shows that inadequate intakes of vitamin D, vitamin K, calcium, niacin, and vitamin B_6_ showed particularly strong associations with GDM risk in women with BMI ≥ 25.0 kg/m^2^, with relative risks exceeding 2.0 for most of these nutrients. In contrast, these associations were generally weaker or non-significant in women with BMI < 25.0 kg/m^2^.

## 4. Discussion

This study provides novel evidence that overall nutritional adequacy during early pregnancy, as measured by the MAR, is inversely associated with GDM risk in Korean pregnant women. Women in the lowest MAR quartile demonstrated 1.82 times higher odds of developing GDM compared to those in the highest quartile, with a significant dose–response relationship. This association was particularly pronounced among obese women, where inadequate nutritional intake was associated with up to 4.20 times higher GDM risk. Additionally, we identified widespread nutrient inadequacies in this population, with over 90% of participants having insufficient vitamin D and magnesium intake, and specific micronutrient deficiencies—particularly vitamin D, vitamin K, vitamin B_6_, niacin, and calcium—showing significant associations with increased GDM risk.

### 4.1. Interpretation of Overall Nutritional Adequacy Findings

Our finding that women with the lowest MAR had a significantly higher risk of GDM compared to those with the highest MAR provides evidence for the protective role of overall nutritional adequacy. These findings align with Looman et al., who reported that Australian women in the highest quartile of pre-pregnancy micronutrient adequacy had a 39% lower GDM risk [28]. However, our study advances this knowledge by (1) demonstrating these associations in an Asian population with distinct dietary patterns, (2) utilizing first-trimester rather than pre-pregnancy assessments, and (3) showing a significant dose–response relationship. This progressive pattern across MAR quartiles supports the growing recognition that overall dietary balance, rather than individual nutrients in isolation, influences metabolic health during pregnancy [29]. The underlying mechanisms likely involve multiple pathways: adequate micronutrient status improves insulin sensitivity, reduces oxidative stress and inflammation, and optimizes placental function, all of which are critical for maintaining glucose homeostasis during pregnancy [30]. Our results are further supported by recent systematic reviews from 2021 and 2025, which confirmed that overall nutritional adequacy, including micronutrients, provides protective effects against GDM and remains central to contemporary GDM management [31,32]. These consistent findings across different time periods validate that the fundamental biological relationships between nutritional adequacy and GDM risk have remained stable.

### 4.2. Specific Micronutrient Inadequacies and GDM Risk

The identification of specific nutrient inadequacies associated with GDM risk provides insights into potential biological mechanisms and intervention targets. Vitamin D inadequacy showed the strongest association with GDM risk in our study, consistent with its well-established role in insulin secretion and sensitivity [33]. Vitamin D receptors are present in pancreatic β-cells, where vitamin D regulates insulin secretion by modulating β-cell function and suppressing adipogenesis, thereby reducing insulin resistance [34]. The extremely high prevalence of vitamin D inadequacy in our population (over 95%) highlights this as a critical public health concern.

Vitamin K inadequacy emerged as another significant risk factor. Vitamin K regulates glucose metabolism through osteocalcin carboxylation, which influences insulin sensitivity [35]. Studies have shown that vitamin K supplementation improves insulin sensitivity in humans [36], making this an important area for future research in pregnancy. The associations observed for B vitamins (vitamin B_6_ and niacin) are biologically plausible given their roles in energy metabolism and cellular function [37,38]. Calcium inadequacy was also significantly associated with GDM risk, as intracellular calcium signaling is crucial for insulin secretion and glucose uptake [39]. Beta-cell adaptation to pregnancy requires increased insulin secretion, a process tightly regulated by calcium dynamics [40].

### 4.3. BMI-Specific Nutritional Vulnerabilities

An important finding of this study is the BMI-specific variation in the relationship between nutritional adequacy and GDM risk, with the strongest association between nutritional inadequacy and increased GDM risk observed in women with BMI ≥ 25.0 kg/m^2^. The heightened vulnerability to nutritional inadequacy in obese women may reflect several mechanisms. Obesity is associated with chronic low-grade inflammation and increased oxidative stress, conditions that increase micronutrient requirements. Obesity has been characterized as a “fat sickness” with low-grade subclinical systemic and tissue-specific inflammation causally linked to insulin resistance [41,42]. Obese individuals often experience relative micronutrient deficiencies despite adequate or excessive energy intake, a phenomenon termed “hidden hunger” [43,44]. The particularly strong associations observed for vitamin D, vitamin K, calcium, niacin, and vitamin B_6_ inadequacies in obese women support this interpretation, as these nutrients play critical roles in insulin signaling, glucose metabolism, and inflammation regulation [42,45]. These findings suggest that obese pregnant women may require more intensive nutritional counseling and monitoring to achieve adequate nutrient intake during early pregnancy.

### 4.4. Implications for Clinical Practice and Public Health

Our findings have several important implications for clinical practice and public health policy. The high prevalence of nutrient inadequacies observed in our study population suggests that current dietary practices among Korean pregnant women are insufficient to meet pregnancy-related nutritional demands. This is particularly concerning given that dietary assessments were conducted during the first trimester, a critical period for fetal development and metabolic programming. The identification of overall nutritional adequacy, as measured by MAR, as a significant predictor of GDM risk suggests that comprehensive dietary assessment and counseling should be integrated into early prenatal care. The BMI-stratified findings suggest that the intensity of nutritional counseling may need to be tailored according to pre-pregnancy BMI status, with obese women potentially requiring more intensive support. Given the widespread inadequacies of specific micronutrients identified in this study, particularly vitamin D, vitamin K, calcium, and B vitamins, public health strategies to improve dietary intake of these nutrients among pregnant women, especially during early pregnancy, warrant consideration.

### 4.5. Strengths and Limitations

This study has several strengths. First, we used a prospective cohort design with dietary assessment conducted during the first trimester, well before GDM diagnosis, minimizing recall bias and reverse causation. Second, we employed standardized nutritional adequacy indices (NAR and MAR) that account for individual nutrient requirements rather than relying solely on absolute intake values [24]. Third, our BMI-stratified analysis provides novel insights into how the relationship between nutritional adequacy and GDM risk varies according to pre-pregnancy body composition. Fourth, the use of the Korean Dietary Reference Intakes ensures that our assessment of nutritional adequacy is appropriate for this population.

However, several limitations should be acknowledged. First, our data were collected between March 2013 and January 2017, representing relatively older data. However, as discussed in the interpretation section above, recent systematic reviews from 2021 and 2025 that examined studies through the most current period continue to confirm that overall nutritional adequacy, including micronutrients, remains central to GDM prevention [31,32]. Second, dietary intake was assessed using a single 24 h dietary recall, which may not fully capture usual dietary patterns or day-to-day variation in intake. However, this method has been validated for use in Korean populations when conducted by trained professionals [22], and we employed visual aids to improve accuracy. Third, while we adjusted for multiple potential confounders, residual confounding from unmeasured factors such as genetic predisposition, gut microbiota composition, or environmental exposures cannot be entirely excluded. Fourth, the observational nature of this study precludes causal inference, and intervention studies are needed to determine whether improving nutritional adequacy can prevent GDM. Fifth, our study population consisted of women attending two hospitals in Seoul, which may limit generalizability to rural populations or women of different socioeconomic backgrounds. Sixth, the relatively small number of GDM cases in some subgroups, particularly in the BMI-stratified analyses, may have limited statistical power to detect associations. Additionally, we did not assess dietary supplement use in detail, which could have influenced total nutrient intake and adequacy. Finally, we collected information on supplement use as a covariate; we did not quantify supplement-derived nutrient intake in our adequacy calculations. This may have resulted in underestimation of total nutrient intake for some participants. Future studies should incorporate comprehensive assessment of both dietary and supplemental nutrient sources.

## 5. Conclusions

This study demonstrates that overall nutritional adequacy during early pregnancy, as measured by MAR, is inversely associated with GDM risk in Korean pregnant women, with particularly strong associations observed in women with BMI ≥ 25.0 kg/m^2^. Specific micronutrient inadequacies, particularly vitamin D, vitamin K, vitamin B_6_, niacin, and calcium, are widespread in this population and significantly associated with increased GDM risk. These findings underscore the importance of comprehensive nutritional assessment and counseling as part of early prenatal care, with particular attention to women with elevated pre-pregnancy BMI. The development of targeted nutritional interventions based on individual adequacy profiles may represent a promising strategy for GDM prevention.

## Figures and Tables

**Table 1:** Definitions and abbreviations of the nutritional assessment terms used in the current study.

Nutritional Assessment Term	Abbreviation	Definition
Estimated Average Requirement	EAR	Daily nutrient intake level needed to meet the requirements of half the healthy individuals in a particular life stage and gender group
Adequate Intake	AI	Average daily nutrient intake level based on observed or experimentally determined approximations or estimates of nutrient intakes by a group of apparently healthy people that are assumed to be adequate
Recommended Dietary Intake	RDI	The average daily dietary intake level that is sufficient to meet the nutrient requirements of nearly all (97–98 percent) healthy individuals in a particular life stage and gender group

**Table 2:** Baseline characteristics of 2227 pregnant women according to quartile of mean adequacy ratio (MAR).

Characteristic	Total (*n* = 2227)	MAR Q1 (*n* = 556)	Q2 (*n* = 557)	Q3 (*n* = 557)	MAR Q4 (*n* = 557)	*p*
Median MAR (range) ^a^		0.62 (0.07–0.68)	0.72 (0.68–0.76)	0.79 (0.76–0.83)	0.87 (0.83–0.99)	
Age, years	33.4 ± 3.8	33.2 ± 3.7	33.3 ± 3.8	33.6 ± 4.0	33.6 ± 3.8	0.34
Educational level						
High school	203	79 (14.1)	47 (8.4)	37 (6.6)	44 (7.8)	<0.001
college	1657	403 (71.7)	424 (75.4)	434 (77.1)	409 (72.8)	
graduate	367	80 (14.2)	91 (16.2)	92 (16.3)	109 (19.4)	
Income ^b^						
<4 million/Korean won	747	205 (36.5)	175 (31.1)	187 (33.2)	191 (34.0)	0.299
≥4 million/Korean won	1480	357 (63.5)	387 (68.9)	376 (66.8)	371 (66.0)	
Employment status						
Employed	1384	336 (59.8)	368 (65.5)	361 (64.1)	328 (58.4)	0.041
Not employed	843	226 (40.2)	194 (34.5)	202 (35.9)	234 (41.6)	
MVPA participation before pregnancy ^c^						
Yes	1342	344 (61.2)	304 (54.1)	356 (63.2)	356 (63.3)	0.004
No	885	218 (38.8)	258 (45.9)	207 (36.8)	206 (36.7)	
MVPA participation in 1st trimester ^c^						
Yes	705	180 (32.0)	158 (28.1)	198 (35.2)	182 (32.4)	0.088
No	1522	382 (68.0)	404 (71.9)	365 (64.8)	380 (67.6)	
Dietary supplement use						
Yes	2166	543 (96.6)	541 (96.3)	553 (98.2)	551 (98.0)	0.099
No	61	19 (3.4)	21 (3.7)	10 (1.8)	11 (2.0)	
Alcohol use						
Yes	1874	481 (85.6)	472 (84.0)	477 (84.7)	459 (81.7)	0.316
No	353	81 (14.4)	90 (16.0)	86 (15.3)	103 (18.3)	
Smoking currently						
Never smoked	1970	478 (85.1)	499 (88.8)	512 (90.9)	502 (89.3)	0.05
Pre-pregnancy smoking	189	58 (10.3)	50 (8.9)	37 (6.6)	45 (8.0)	
Smoking in early pregnancy ^d^	68	26 (4.6)	13 (2.3)	14 (2.5)	15 (2.7)	
Parity						
0	1313	353 (62.8)	341 (60.7)	319 (56.7)	312 (55.5)	0.044
≥1	914	209 (37.2)	221 (39.3)	244 (43.3)	250 (44.5)	
History of Diabetes Mellitus						
Yes		6 (1.1)	7 (1.2)	5 (0.9)	4 (0.7)	0.821
No	2227	556 (98.9)	555 (98.8)	558 (99.1)	558 (99.3)	
Nausea and vomiting of pregnancy						
No	446	89 (15.8)	127 (22.6)	115 (20.4)	125 (22.2)	0.083
Mild NVP	1373	363 (64.6)	331 (58.9)	345 (61.3)	345 (61.4)	
Severe NVP	408	110 (19.6)	104 (18.5)	103 (18.3)	92 (16.4)	
Pre-pregnancy BMI category						
<18.5 kg/m^2^	296	72 (12.8)	67 (11.9)	86 (15.3)	71 (12.6)	0.457
18.5–22.9 kg/m^2^	1454	348 (61.9)	371 (66.0)	365 (64.8)	375 (66.7)	
23.0–24.9 kg/m^2^	227	64 (11.4)	61 (10.9)	53 (9.4)	56 (10.0)	
≥25.0 kg/m^2^	250	78 (13.9)	63 (11.2)	59 (10.5)	60 (10.7)	
Hypertension						
Yes	33	14 (2.5)	7 (1.2)	6 (1.1)	11 (2.0)	0.221
No	2194	548 (97.5)	555 (98.8)	557 (98.9)	551 (98.0)	
Gestational Diabetes Mellitus						
Yes	157	49 (8.8)	45 (8.1)	34 (6.1)	29 (5.2)	0.066
No	2070	507 (91.2)	512 (91.9)	523 (93.9)	528 (94.8)	
Total energy intake (kcal/day)	1729.0 ± 492.0	1327.8 ± 366.4	1663.8 ± 384.7	1820.0 ± 383.1	2103.6 ± 475.5	<0.001
Total carbohydrate intake (E%)	58.5 ± 10.4	60.6 ± 13.6	58.7 ± 9.6	58.6 ± 8.6	56.2 ± 8.5	<0.001
Total protein intake (E%)	16.4 ± 6.0	15.7 ± 10.8	15.9 ± 3.0	16.6 ± 2.9	17.5 ± 3.0	<0.001

MAR, Mean Adequacy Ratio; MVPA, Moderate-to-Vigorous Physical Activity; BMI, Body Mass Index. Variables are expressed as mean ± standard deviation or as a number (%) of cases. The *p*-value was obtained using ANOVA or chi-square statistical analysis. ^a^ Calculated as the mean of individual Nutrient Adequacy Ratios (NARs) for selected nutrients. NAR for each nutrient was computed as (individual intake/recommended intake) × 100, truncated at 100 to prevent overestimation from excessive intake of single nutrients. ^b^ Reflecting the Korean household average monthly income in 2013. ^c^ Moderate-to-vigorous physical activity ≥ 150 min per week, assessed using International Physical Activity Questionnaire. ^d^ Continued to smoke from before pregnancy through study enrollment (early pregnancy).

**Table 3:** Odds ratio (95% CIs) for association between mean nutrient adequacy ratio and incidence of gestational diabetes (*n* = 2227).

	Quartiles of Mean Adequacy Ratio				*p* for Trend ^d^
	Quartile 1	Quartile 2	Quartile 3	Quartile 4	
Median MAR	0.62	0.72	0.79	0.87	
GDM cases/Normal cases	49/507	45/512	34/523	29/528	
Model 1 ^a^	1.760 (1.080–2.867)	1.719 (1.049–2.816)	1.242 (0.738–2.092)	1.00 (ref) ^c^	0.013
Model 2 ^b^	1.815 (1.100–2.994)	1.750 (1.061–2.884)	1.270 (0.753–2.143)	1.00 (ref) ^c^	0.012

CI, Confidence Intervals; MAR, Mean Adequacy Ratio; GDM, Gestational Diabetes Mellitus. ^a^ Odds ratios were obtained using logistic regression model adjusted for age, pre-pregnancy BMI, employment status, parity, and hypertension. ^b^ Model 1 + additional adjustment for carbohydrate (E%), protein (E%), saturated fat (E%) and fiber (g/d). ^c^ reference group. Women in Quartile 4 (highest nutritional adequacy) served as the reference category with OR = 1.00, against which the GDM risk for women in lower quartiles (Q1–Q3) was compared. ^d^ *p* for trend was calculated by including the median MAR value of each quartile as a continuous variable in the logistic regression model.

**Table 4:** Nutrient intake, prevalence of inadequate nutrient intake and relative risks (95% CIs) for associations between inadequate nutrient intakes and incidence of gestational diabetes (*n* = 2227).

Dietary Intake	EAR (AI)	Normal (*n* = 2070)	GDM (*n* = 157)	*p*	Normal (*n* = 2070)	GDM (*n* = 157)	Chi-Squared	Relative Risk
		Mean (SD)	Mean (SD)		% Below EAR/AI ^a^	% Below EAR/AI ^a^		
Energy, kcal/d	2000–1900 *	1730.9 ± 486.5	1703.0 ± 559.8	0.492	68.7	70.7	0.610	1.09 (0.78–1.52)
Energy-adjusted carbohydrate, g/d	135	280.7 ± 44.4	277.3 ± 43.1	0.347	4	3.2	0.628	0.81 (0.34–1.92)
Energy-adjusted lipid, g/d	-	62.1 ± 52.1	62.2 ± 16.9	0.985				
Energy-adjusted protein, g/d	45–40	80.6 ± 24.2	79.5 ± 16.1	0.570	6.7	9.6	0.176	1.42 (0.86–2.36)
Dietary fiber, g/d	(25)	20.7 ± 7.2	20.2 ± 7.6	0.431	76.8	77.7	0.798	1.05 (0.73–1.51)
Vitamin A, μg RAE/d	510–500	506.4 ± 323.7	469.9 ± 265.4	0.169	57.9	62.4	0.266	1.19 (0.87–1.63)
Thiamin, mg/d	1.3	1.3 ± 0.7	1.3 ± 0.7	0.971	58.4	59.2	0.839	1.03 (0.76–1.40)
Riboflavin, mg/d	1.3	1.3 ± 0.6	1.2 ± 0.5	0.315	58.6	61.1	0.532	1.10 (0.81–1.50)
Niacin, mg NE/d	14	16.4 ± 10.3	15.2 ± 6.8	0.164	40.1	51.6	0.005	1.54 (1.14–2.08)
Vitamin B_6_, mg/d	1.9	1.6 ± 0.7	1.5 ± 0.6	0.040	74.2	82.8	0.017	1.62 (1.08–2.43)
Folate, μg DFE/d	340	560.7 ± 213.5	526.4 ± 200.7	0.052	46.9	54.8	0.057	1.34 (0.99–1.82)
Vitamin B12, μg/d	2.2	9.0 ± 6.6	7.9 ± 6.1	0.062	6.6	8.9	0.258	1.36 (0.80–2.29)
Vitamin C, mg/d	85	118.5 ± 77.0	104.1 ± 66.8	0.022	41.1	48.4	0.074	1.32 (0.97–1.78)
Vitamin D, μg/d	(10)	4.01 ± 5.3	3.6 ± 3.2	0.087	92.7	98.1	0.010	3.84 (1.24–11.90)
Vitamin E, mg α-TE/d	(12)	16.9 ± 7.5	16.1 ± 7.3	0.212	26.8	31.2	0.232	1.22 (0.88–1.69)
Vitamin K, μg/d	(65)	274.6 ± 204.2	262.3 ± 207.0	0.466	5.0	9.6	0.014	1.89 (1.15–3.11)
Calcium, mg/d	550	557.0 ± 250.3	535.8 ± 271.1	0.310	55.1	63.7	0.037	1.39 (1.02–1.91)
Iron, mg/d	19	14.3 ± 5.5	13.6 ± 5.2	0.106	85.4	86.6	0.677	1.10 (0.71–1.71)
Magnesium, mg/d	260–270	81.1 ± 49.1	72.6 ± 43.7	0.035	99.6	100	0.435	-
Selenium, μg/d	53	97.3 ± 36.1	92.3 ± 35.4	0.092	7.7	11.5	0.096	1.49 (0.94–2.38)
Phosphorus, mg/d	580	1113.9 ± 354.3	1072.7 ± 362.3	0.161	3.8	6.4	0.107	1.65 (0.90–3.03)
Zinc, mg/d	9	10.0 ± 3.5	9.8 ± 3.6	0.610	42.0	46.5	0.275	1.18 (0.88–1.60)
Copper, μg/d	600	1159.1 ± 401.2	1146.7 ± 436.1	0.712	4.2	6.4	0.200	1.49 (0.81–2.74)
Sodium, mg/d	(3000)	4279.4 ± 1468.8	4267.3 ± 1729.5	0.932	19.7	26.1	0.054	1.40 (0.99–1.97)
Potassium, mg/d	(3500)	2917.7 ± 1019.7	2736.5 ± 874.5	0.030	76.4	82.2	0.100	1.39 (0.94–2.07)

CI, Confidence Intervals; GDM, Gestational Diabetes Mellitus; EAR, Estimated Average Requirement; AI, Adequate intake; SD, Standard Deviation; RAE, Retinol Activity Equivalents; NE, Niacin Equivalents; DFE, Dietary Folate Equivalents; α-TE, α-Tocopherol Equivalents. * Ranges indicate different values for age groups 19–29 and 30–49 years. Continuous variables were compared using independent *t*-tests and categorical variables using chi-square tests. ^a^ Proportion of individuals with nutrient intake below the EAR, or below the AI when EAR is not established.

**Table 5:** Odds ratio (95% CIs) for the association between average nutrient adequacy ratio, stratified by body mass index, and incidence of gestational diabetes (*n* = 2227).

Subgroup		Quartiles of Mean Adequacy Ratio				*p* for Trend ^b^
		Quartile 1	Quartile 2	Quartile 3	Quartile 4	
	Median MAR	0.62	0.72	0.79	0.87	
BMI ≤ 22.9 kg/m^2^ (*n* = 1730)	GDM/Normal ^a^	24/395	25/414	20/429	17/426	
	Model ^c^	1.96 (1.01–3.79)	1.86 (0.97–3.57)	1.31 (0.67–2.57)	1.00 (ref) ^d^	0.031
BMI 23.0–24.9 kg/m^2^ (*n* = 227)	GDM/Normal	7/55	5/53	3/49	7/48	
	Model ^c^	0.87 (0.26–2.92)	0.62 (0.17–2.21)	0.40 (0.10–1.68)	1.00 (ref)	0.999
BMI ≥ 25.0 kg/m^2^ (*n* = 250)	GDM/Normal	18/57	15/45	11/45	5/54	
	Model ^c^	3.17 (0.99–10.15)	4.20 (1.30–13.50)	2.64 (0.81–8.58)	1.00 (ref)	0.037

CI, Confidence Intervals; MAR, Mean Adequacy Ratio; GDM, Gestational Diabetes Mellitus. ^a^ Number of GDM Cases/Normal Cases. ^b^ *p* for trend was calculated by including the median MAR value of each quartile as a continuous variable in the logistic regression model. ^c^ Odds ratios were obtained using logistic regression model adjusted for age, employment status, parity, and hypertension, carbohydrate (E%), protein (E%), saturated fat (E%) and fiber (g/d). ^d^ reference group. Women in Quartile 4 (highest nutritional adequacy) served as the reference category with OR = 1.00, against which the GDM risk for women in lower quartiles (Q1–Q3) was compared.

## Data Availability

The data presented in this study are available on request from the corresponding author due to patient privacy protection.

## Abbreviations

AI	Adequate Intake
ANOVA	Analysis of Variance
BMI	Body Mass Index
CAN-Pro	Computer Aided Nutritional Analysis Program
CI	Confidence Interval
DASH	Dietary Approaches to Stop Hypertension
EAR	Estimated Average Requirement
GCT	Glucose Challenge Test
GDM	Gestational Diabetes Mellitus
HEI	Healthy Eating Index
IPAQ	International Physical Activity Questionnaire
KPOS	Korean Pregnancy Outcome Study
MAR	Mean Adequacy Ratio
MVPA	Moderate-to-Vigorous Physical Activity
NAR	Nutrient Adequacy Ratio
OGTT	Oral Glucose Tolerance Test
OR	Odds Ratio
RDI	Recommended Dietary Intake
RR	Relative Risk

## Appendix A

**Table A1:** Baseline characteristics by GDM status (*n* = 2227).

Characteristic	Total (*n* = 2227)	Normal (*n* = 2070)	GDM (*n* = 157)	*p*
Age, years	33.4 ± 3.8	33.3 ± 3.8	35.0 ± 3.6	<0.001
Educational level				
High school	203	182 (8.8)	21 (13.4)	0.070
college	1657	1540 (74.4)	117 (74.5)	
graduate	367	345 (16.8)	19 (12.1)	
Income ^a^				
<4million/Korean won	747	685 (33.1)	62 (39.5)	0.102
≥4million/Korean won	1480	1385 (66.9)	95 (60.5)	
Employment status				
Employed	1384	1300 (62.8)	84 (53.5)	0.021
Not employed	843	770 (372)	73 (46.5)	
MVPA at pre-pregnancy ^b^				
Yes	1342	1252 (60.5)	90 (57.3)	0.436
No	885	818 (39.5)	67 (42.7)	
MVPA at 1st trimester ^b^				
Yes	705	658 (31.8)	47 (29.9)	0.631
No	1522	1412 (68.2)	110 (70.1)	
Dietary supplement use				
Yes	2166	2015 (97.3)	151 (96.2)	0.389
No	61	55 (2.7)	6 (3.8)	
Balanced dietary intake status				
Yes	1157	1092 (52.8)	65 (41.4)	0.006
No	1070	978 (47.2)	92 (58.6)	
Alcohol use				
Yes	1874	1742 (84.2)	132 (84.1)	0.979
No	353	328 (15.8)	25 (15.9)	
Smoking currently				
Never smoked	1970	1838 (88.8)	132 (84.1)	0.086
Pre-pregnancy smoking	189	173 (8.4)	16 (10.2)	
Smoking in early pregnancy ^c^	68	59 (2.9)	9 (54.7)	
Parity				
0	1313	1236 (59.7)	77 (49.0)	0.009
≥1	914	834 (40.3)	80 (51.0)	
History of DM				
Yes		-	-	-
No	2227	2070 (100)	157 (100)	
Nausea and vomiting of pregnancy				
No	446	424 (20.5)	22 (14.0)	0.080
Mild NVP	1373	1274 (61.5)	99 (63.1)	
Severe NVP	408	372 (18.0)	36 (22.9)	
Pre-pregnancy BMI category				
<22.9 kg/m^2^	1750	1664 (80.4)	86 (54.8)	<0.001
23.0–24.9 kg/m^2^	227	205 (9.9)	22 (14.0)	
≥25.0 kg/m^2^	250	201 (9.7)	49 (31.2)	
Hypertension				
Yes	33	27 (1.3)	6 (3.8)	0.012
No	2194	2043 (98.7)	151 (96.2)	

MVPA, Moderate-to-Vigorous Physical Activity; BMI, Body Mass Index. Variables are expressed as mean ± standard deviation or as a number (%) of cases. The *p*-value was obtained using *t*-test or chi-square statistical analysis. ^a^ Reflecting the Korean household average monthly income in 2013. ^b^ Moderate-to-vigorous physical activity ≥150 min per week, assessed using International Physical Activity Questionnaire. ^c^ Continued to smoke from before pregnancy through study enrollment (early pregnancy).
